# Outcomes in Economic Evaluations of Public Health Interventions in Low‐ and Middle‐Income Countries: Health, Capabilities and Subjective Wellbeing

**DOI:** 10.1002/hec.3302

**Published:** 2016-01-25

**Authors:** Giulia Greco, Paula Lorgelly, Inthira Yamabhai

**Affiliations:** ^1^ London School of Hygiene & Tropical MedicineLondonUK; ^2^ Centre for Health EconomicsMonash UniversityMelbourneVictoriaAustralia; ^3^ Health Intervention and Technology Assessment Program (HITAP)Ministry of Public HealthBangkokThailand

**Keywords:** capabilities, subjective wellbeing, happiness, life satisfaction, economic evaluation, public health

## Abstract

Public health programmes tend to be complex and may combine social strategies with aspects of empowerment, capacity building and knowledge across sectors. The nature of the programmes means that some effects are likely to occur outside the healthcare sector; this breadth impacts on the choice of health and non‐health outcomes to measure and value in an economic evaluation.

Employing conventional outcome measures in evaluations of public health has been questioned. There are concerns that such measures are too narrow, overlook important dimensions of programme effect and, thus, lead to such interventions being undervalued. This issue is of particular importance for low‐income and middle‐income countries, which face considerable budget constraints, yet deliver a large proportion of health activities within public health programmes.

The need to develop outcome measures, which include broader measures of quality of life, has given impetus to the development of a variety of new, holistic approaches, including Sen's capability framework and measures of subjective wellbeing. Despite their promise, these approaches have not yet been widely applied, perhaps because they present significant methodological challenges. This paper outlines the methodological challenges for the identification and measurement of broader outcomes of public health interventions in economic evaluation in low‐income and middle‐income countries.

## 1 Public Health and Its Outcomes

Public health interventions aim to protect health, prolong life and improve the lives of people (Weatherly *et al*., 2009, Kickbusch and Nutbeam, 1998, Rychetnik *et al*., 2002). The ‘new public health’ paradigm (Tulchinsky and Varavikova, 2014) has moved public health into the health promotion era (Awofeso, 2004), whereby health promotion activities seek to initiate or increase community participation in order for individuals to improve their health through attitudinal, behavioural, social and environmental changes. Much of this paper will focus on public health as health promotion, but the terms may be used interchangeably. Public health in many low‐income and middle‐income countries (LMICs) is still in the era of preventive health care (seeking to improve public health by focusing on the prevention and treatment of diseases in high‐risk groups; e.g. vaccination campaigns) or primary health (‘Health for All’ – effective health care for the community; e.g. universal health care) (Awofeso, 2004). But public health of the health promotion type is growing in its application in LMICs, and there will soon be a need to show its value.

Public health programmes tend to be complex and may often combine educational, social and political strategies with aspects of empowerment, capacity building and knowledge across different sectors (Kickbusch and Nutbeam, 1998, Borghi and Jan, 2008). For example, not only is the MaiMwana project, a community‐based maternal and child health programme in Malawi, focused on improving the health of mother and child, but it also seeks to empower women, build capacity and impart knowledge (Lewycka *et al*., 2013). Many of the effects of public health programmes are likely to occur outside the healthcare sector. The breadth of effects will impact on the choice of outcomes, both health and non‐health, which require measurement and, if an economic evaluation is included, may require valuation (Weatherly *et al*., 2009). These outcomes can vary in scope, from changes in the health status of an individual to changes in their behaviour and the behaviour and/or characteristics of communities, to changes in the political and social environment.

For many researchers, the focus of public health programmes and their evaluation continues to be the maximisation of health, perhaps because of our history with health technology assessment; this means that non‐health outcomes are often ignored (Borghi and Jan, 2008). However, neglecting to consider multiple effects might overlook potentially important benefits, and the holistic impact of the programme could be underestimated and undervalued. For example, a health promotion programme in Nepal whereby women's groups were used to improve birth outcomes in rural areas found by using contingent valuation methods that 84% of respondents valued the non‐health outcomes – identified as increased knowledge, confidence and social participation – compared with 56% who valued the health outcomes (42% stated they valued both health and non‐health outcomes); therefore, focusing on health outcomes alone would undervalue the programme (Borghi and Jan, 2008). Rychetnik *et al*. (2002) argue that the evaluation of the effectiveness of public health interventions must be sufficiently comprehensive to cover their complexity, and the evaluative space should be extended in order to include health and non‐health outcomes within an adequate time frame.

Cost‐effectiveness analysis and cost–utility analysis are now a standard part of evidence generation for clinical interventions; economic evaluations of public health programmes are increasing, but their application presents substantial methodological challenges (Borghi and Jan, 2008, Lorgelly *et al*., 2010, Hale, 2000). Particularly with respect to health promotion, health economists have been somewhat sidelined in the past in undertaking economic evaluations. Hale (2000) argued that this was due to a lack of demand, misunderstanding by health promotion activists of the contribution of health economics, misunderstanding by health economists of what health promotion is and aims to do, and perhaps, most importantly, technical difficulties in applying standard methods of economic evaluation to health promotion interventions. Many authors have acknowledged the technical difficulties that economists face in capturing and evaluating both the costs and the benefits of these complex interventions (Lorgelly *et al*., 2010, Shiell and Hawe, 1996, Rosen and Lindholm, 1992, Dixon and Sindall, 1994, Hale, 2000).

Weatherly *et al*. (2009) and Chalkidou *et al*. (2008) both present a list of methodological challenges faced when undertaking evaluations of public health programmes; common to each is the measurement and valuation of the outcomes of public health programmes and, specifically, the need for multiple outcome measures or broader outcome measures.

This present paper discusses this issue with respect to undertaking evaluations of public health programmes in LMICs; readers interested in a general comparison of economic evaluations across the development spectrum are directed to Griffiths and others (2016) in this special issue. This paper proceeds by first reviewing the standard approaches used to identify, measure and value outcomes in the assessment of cost‐effectiveness and their limitations to both public health programmes and the LMIC setting. We then discuss two new approaches, the capability approach and subjective wellbeing (SWB), as means to assess outcomes in this setting; included in this presentation are exemplar case studies from LMICs where these approaches have been employed. We conclude with a discussion of remaining and outstanding issues, many of which are not unique to LMICs, but may be more challenging in this setting.

## 2 Standard Approaches to Measuring and Valuing Outcomes

There are four types of economic evaluations that are available in a researcher's toolkit to evaluate public health programmes: a cost consequences analysis (CCA), a cost‐effectiveness analysis (CEA), a cost–utility analysis (CUA) and a cost–benefit analysis (CBA). Table 1 describes each type in detail and also presents the advantages, disadvantages and value positions of adopting one type of evaluation or another. Specific to this paper is the nature of the outcomes that each type utilises.

**Table 1:** Types of economic evaluations: measurement of outcomes, advantages and disadvantages (adapted from Drummond, 2007, p. 100)

Cost consequences analysis	
Outcome	Natural units (e.g. life years gained, disability days saved and points of blood pressure reduction)
	Can include non‐health outcomes
	Includes multiple outcomes
	**Advantages**
Value position	Not defined. Flexibility because decision maker can apply own decision rules
Practical feasibility	A broad scope of outcomes can be measured including non‐health and health outcomes. Outcomes are presented in a disaggregated manner so that the benefits and disbenefits associated with each intervention are reported upfront. This can aid transparency.
	**Disadvantages**
Value position	No theoretical basis
Practical feasibility	Lack of transparency in terms of decision rules. Decision maker applies own subjective decision rules about the trade‐offs between different outcomes and the trade‐off between outcomes and costs
Cost‐effectiveness analysis	
Outcomes	Natural units (e.g. life years gained, disability days saved, points of blood pressure reduction and DALYs)
	**Advantages**
Value position	Underpinned by extra‐welfarism, incorporating the objective of maximising health; although only addresses technical efficiency
Practical feasibility	Standardised measurement in natural units (DALY weights are also standard)
	**Disadvantages**
Value position	Not consistent with traditional welfare economics as the objective is to maximise health rather than subjective utility
Practical feasibility	By focusing on health outcomes, the approach omits non‐health outcomes. DALYs have fixed weights irrespective of the population.
Cost–utility analysis	
Outcomes	Healthy years typically measured as quality‐adjusted life years
	**Advantages**
Value position	Health state preferences can be elicited using choice based preferences, that is, either standard gamble utilities or time‐trade‐off values
	Can incorporate preferences of the general public ‘behind a veil of ignorance’, consistent with the Rawlsian theory
	Life years are adjusted for the quality of those life years
Practical feasibility	Underpinned by extra‐welfarism, incorporating the objective of maximising health; although only addresses technical efficiency
	**Disadvantages**
Value position	Not consistent with traditional welfare economics as the objective is to maximise health rather than subjective utility
Practical feasibility	By focusing on health outcomes, the approach omits non‐health outcomes
	Different health state valuation tools can generate different valuations for the same health state
Cost–benefit analysis	
Outcomes	Monetary units based on individual compensation
	**Advantages**
Value position	Consistent with traditional welfare economics incorporating objective of maximising individual subjective utility. Decision rule: if benefits > costs, the social welfare is greater
Practical feasibility	Broad scope of outcomes can be measured in monetary values including non‐health as well as health outcomes. Non‐health outcomes include process utility, for example, the reassurance value associated with conducting diagnostic tests
	**Disadvantages**
Value position	WTP values may be influenced by individuals' ability to pay. Although it can be adjusted for
Practical feasibility	WTP elicitation has been associated with issues of bias and precision
	Insensitive to the magnitude of effect including scope effects and nesting effects
	Inflate valuations of the specific intervention that respondents are asked about, relative to interventions that respondents are not asked about
	Difficult to validate WTP if public health care is free at the point of delivery
	Lack of standardised elicitation process: different question formats used can yield different results. For example, payment card bidding approach compared with dichotomous choice take‐it‐or‐leave‐it approach

DALY, disability adjusted life year; WTP, willingness‐to‐pay.

A cost consequences analysis is the simplest of evaluations: costs are compared with outcomes, which are presented in a disaggregated tabular way (Coast, 2004). The approach can be useful when mapping the intersectoral costs and multiple outcomes of public health interventions (Lorgelly *et al*., 2010). However, as the evaluation explicitly does not combine the information (there is no suggestion that ratios comparing cost with each outcome should be presented), then it can be problematic when informing allocation decisions, as the value position is left open to the decision makers and is often implicit (Weatherly *et al*., 2009).

Because of the need for evidence‐based decision making and explicit decisions, CEAs, where incremental costs are compared with incremental outcomes (in the form of an incremental cost‐effectiveness ratio (ICER)), are the most common type of economic evaluation (for both health technology assessment and the evaluation of public health programmes) (McDaid and Needle, 2008). The use of natural units of outcome, as is often the case with a CEA, is, however, only informative when comparing like with like. For example, if choosing between multiple maternal and child health programmes, then it should be possible to compare the cost per infant death averted or some other common outcome (and choose the programme that produces the greatest gain for the least cost, the smallest ICER), but if the budgeting decision is to also consider an HIV education campaign, then the outcomes of this programme (infections avoided) are not directly comparable with those of the maternal and child health programme. In LMIC settings, this comparability issue can be overcome by using disability‐adjusted life years (DALYs) and estimating cost per DALYs averted. In DALY measurement, health states are described in clinical terms. The value of a health state is determined by their ‘disability weight’; this weight was estimated using a household survey in five countries and an open‐access online survey, where paired comparison questions asked participants to indicate which of two health scenarios represented a greater level of overall health (Salomon *et al*., 2013). The notion of disability describes the impact of impairment on health, and the weights reflect how affected a person's health would be with respect to the given disability; the specific focus is ‘health loss’ rather than ‘welfare loss’, where the former focuses on changes in wellbeing due to physical and mental health, while the latter could include such factors as social considerations that alter wellbeing (Chen *et al*., 2015). With respect to public health interventions and health promotion programmes with broad outcomes, DALYs do not offer a measure of quality of life beyond health/disability. Additional issues with the disability weights are discussed by Nord (2013). Note that there is some debate as to whether DALY weights are utilities, and therefore are more appropriately described as CUAs. DALY weights have been derived using the person trade‐off technique, which Drummond *et al*. (2005) describe as not being preferences; we have chosen to follow this convention, thereby classifying cost per DALYs as a CEA. Note, however, the CUAs are special types of CEAs (Drummond *et al*., 2005).

Growing in popularity in both LMIC and evaluations of public health programmes more generally (McDaid and Needle, 2008) is the application of CUA. CUAs employ quality‐adjusted life years (QALYs), where the quality weight or utility is generally regarded as a measure of health‐related quality of life (HRQoL). The weights are derived using preference elicitation exercises, which now commonly form the basis of a generic multi‐attribute utility instrument, like the EQ‐5D or SF‐6D. The preference elicitation exercises typically involve trading time in a health state or chancing death and a health state, and it is not known if non‐health outcomes influence the elicited preferences of respondents to be in a health state, although it has been shown that while formulating their preferences respondents do take into account aspects of wellbeing outside health (e.g. age, religion and family) (Baker and Robinson, 2004). However, the inferred utility value is not a direct measure of wellbeing *per se* (Dolan *et al*., 2008). Moreover, the QALY framework may not be able to detect small changes in health states, as might happen for community‐based health promotion interventions that generally target healthy individuals (Rosen and Lindholm, 1992).

Weatherly *et al*. (2009) argue that analyses based on either DALYs or QALYs are not well suited to the assessment of public health and community‐based initiatives; because they focus on health, they fail to capture adequately the full range and breadth of outcomes generated by these complex interventions. Furthermore, their common underlying assumption is that health is valued in terms of its impact on wellbeing; thus, they only measure the health‐related part of wellbeing (Bognar, 2008). Many authors have stressed the shortcomings of the QALY in this context and have suggested the use of other types of evaluations (Borghi and Jan, 2008, Rosen and Lindholm, 1992, Hale, 2000).

The final type of evaluation that is available to researchers is a CBA. CBAs are, however, uncommon irrespective of the context, high income or LMIC, public health or clinical intervention; this in part is because outcomes are valued using a monetary metric, which can be fraught with problems (refer to subsequent discussions), although in the context of public health programmes, monetary values are a possible approach to capturing the broad benefits of a public health intervention. Both health and non‐health outcomes generated can be monetised with contingent valuation methods and discrete choice experiments (Ryan, 1999). As discussed earlier, willingness‐to‐pay (WTP) estimates have been elicited for community‐based maternal health interventions in Nepal (Borghi *et al*., 2005) and also in Malawi (Colbourn, 2012). There are a number of criticisms related to the adoption of contingent valuation methods. It has been shown that there is a strong relationship between income and WTP: people with a low income are likely to provide low valuations. When evaluating public health interventions, this could be an issue because many interventions are targeted at low‐income individuals, and the use of WTP could undervalue the real benefit of these interventions. Moreover, the adoption of this compensation mechanism is not always appropriate as people might feel uncomfortable placing a monetary value on a healthy life (Coast, 2004), and the ‘price’ could be biased by the ability to pay of the participant and distorted towards the wealthy (Lorgelly *et al*., 2010), although equity weights can be applied and the distribution can be adjusted subsequently (Borghi, 2008). Moreover, stated preferences can be insensitive to the magnitude of the benefits, for example, a vaccination programme, which offers benefits to others in terms of herd immunity, may be undervalued by individuals receiving the vaccination (Drummond *et al*., 2005).

Attempts to use any of the aforementioned techniques in the economic appraisal of public health and community participatory interventions are generally met with criticism (Smith and Petticrew, 2010, Lorgelly *et al*., 2010, Drummond *et al*., 2008). The main reason is that the full range of outcomes is not incorporated in the appraisal, and hence, the results routinely devalue these types of interventions.

In parallel, there is a wider discussion amongst both academics and policymakers over new measures of wellbeing and social progress, which created a global ‘beyond GDP’ movement (Stiglitz, 2009). The UK government is committed to ‘developing wider measures of wellbeing so that government policies can be more tailored to the things that matter’ (Matheson, 2011, p.9) and is leading the change with the establishment of a ‘What Works’ centre on wellbeing. There is thus a call for developing and testing new methods to measure wellbeing from within both health economics and the wider economic and political sphere.

## 3 New Approaches to Measuring and Valuing Outcomes

The need to develop outcome measures that include multidimensional measures of quality of life and valuation methods, which include social values and a broader participatory process, has given impetus to the development and, in some instances, the adoption of a variety of new approaches. Two main approaches to the measurement of wellbeing have thus far gained traction: the capability approach and SWB.

### 3.1 The capability approach

Theoretical advances in economics, such as Sen's concept of capability, entail a broader notion of wellbeing and have been suggested to be well suited for assessing public health interventions and community development programmes (Mooney, 2005, Lorgelly *et al*., 2010, Smith *et al*., 2012). As Sen states, when assessing quality of life, the object of the assessment should be people's capabilities, intended as the real freedom that people have to live the life they value (Sen, 1985, 1992, 1993).

A crucial normative argument of Sen's capability approach is that quality of life should not be measured as opulence or utility and should not be assessed using people's preferences or desires but should concern people's capabilities: the abilities to achieve those ‘beings and doings’ that people have reason to value in life (Sen, 1993). These valuable ‘beings and doings’ can range from basic functionings, such as being healthy and living in a decent house, to more complex functionings, such as being respected and feeling in control over personal decisions.

The first studies rooted in the capability approach were carried out by Sen himself (Sen, 1985). One of these, which led to the development of the Human Development Index, showed that ranking countries based on income leads to very different results from ranking according to specific functionings.

Sen's approach is not new to health economics, as it formed the basis of Culyer's contribution to the progress of extra‐welfarism and the subsequent development of the QALY framework (Culyer, 1990, Brouwer and Koopmanschap, 2000). However, it is only recently that the capability approach has started to be considered more directly as an alternative to conventional approaches (Cookson, 2005, Coast *et al*., 2008b, Smith *et al*., 2012), and there are now a number of researchers who have operationalised the approach as an outcome measure for use in economic evaluations.

The most widely known outcome measure for use in economic evaluation is the ICEpop CAPability (ICECAP) suite of measures developed at the University of Birmingham (Coast *et al*., 2008a, Al‐Janabi *et al*., 2012). These measures for adults, older people and those at end of life are increasingly used but as yet have not been validated for use in a low‐income setting.

Earlier work comparing measures of capability identified from questions in the British Household Panel Survey with life satisfaction (Anand *et al*., 2009) was developed into a questionnaire and then further refined (using mixed methods) by a group of researchers from the University of Glasgow (Lorgelly *et al*., 2008). They constructed a capability index without undertaking a valuation exercise and instead assigned equal weight to each capability; the OCAP‐18 was found to compare well with both global wellbeing and HRQoL measures (Lorgelly *et al*., 2015). Simon *et al*. (2013) have further adapted the approach; the instrument, known as the OxCAP‐MH, measures outcomes in mental health.

The capability approach is fundamentally a development paradigm; yet to date, there is only one known application to measure capabilities using an existing instrument and only one project that has specifically developed a capability instrument in an LMIC setting.

#### 3.1.1 Capability measurement in an LMIC setting: case study in Thailand

The AIDS Competence Process (ACP), an initiative supported by the Asian Development Bank, aims to enhance the local response to HIV/AIDS with the belief that communities have the capacity to solve their own problems (Constellation, 2012). The project aims to build the capacity of non‐governmental organisations to develop and implement competence within communities for sharing and learning. The project involved partners from Cambodia, India, Indonesia, the Philippines, Papua New Guinea and Thailand.

The approach has been shown to be effective in empowering and mobilising communities to respond to the HIV/AIDS epidemic (UNAIDS/UNITAR, 2005, Morea *et al*., 2009). However, additional evidence, in the form of an economic evaluation, was required by The Constellation and its partners, UNAIDS and the HIV and AIDS Alliance, as to why more (or less) resources should be directed towards the ACP.

In the modelled economic evaluation, Teerawattananon and Yamabhai (2010) assessed the effectiveness of the ACP in terms of change in individual capability, the affect it has on quality of life and HIV risk behaviours and ultimately QALYs (see further discussion in Section 3.1.3 regarding the use of QALYs). The OCAP‐18 questionnaire (Lorgelly *et al*., 2008) was translated into Thai and tested with a sample of the general population in Samut Songkhram Province. The revised questionnaire was used to collect information on personal background, the capability index, quality of life using a visual analogue scale, HIV/AIDS awareness and attitudes and HIV risk factors, from general and high‐risk populations (i.e. men who have sex with men) in Nakhon Nayok and Chiang Mai Provinces.

The study hypothesised that ACP would increase individual capabilities, which would reduce HIV risky behaviour and eventually reduce HIV infections, and that the decreased incidence of HIV infections would result in QALYs being saved. The survey results confirmed that the capability index had a positive relationship with quality of life and a negative relationship with HIV risk, specifically increasing the capability index (on a 0 to 18 scale) by one, increased quality of life (on a 0 to 100 scale) by 4.27, while a one unit increase in capability decreased the relative risk of HIV in the highest‐risk group by around 0.51. These values were used in a decision analytic model in order to attribute the ACP to QALYs (note the increase in QALYs via the quality of life scale was equated to 0.04 on the utility (0 to 1) scale). Additionally, the model included parameters for incidence and cost. The estimated ICER was $US33 to $US37 per QALY ($US1 = 32 baht, 2010). Sensitivity analysis found that ACP is likely to be very cost‐effective (according to the cost‐effectiveness threshold of GDP per capita) compared with existing HIV prevention programmes in Thailand.

#### 3.1.2 Capability measurement in an LMIC setting: case study in Malawi

Greco (2013) has developed an index of capabilities to assess women's quality of life in rural Malawi. The index, which has been validated and can readily be used in similar contexts, is grounded on women's values: the list of capabilities and their weightings are drawn from the bottom up.

Through a series of focus groups in Mchinji district, Malawi, women of childbearing age discussed what a ‘good life’ meant for them, what the valuable components of a good life were and the factors that could enable them or deter them from achieving a good life (Greco *et al*., 2015). After probing by a skilled moderator and lively debating, the women came up with a list of life dimensions expressed as ‘beings, doings, and havings’; subsequently, they placed a value from 0 to 10 to each to indicate a dimension's importance.

The valuable dimensions, or capabilities, for women in rural Malawi are physical strength, inner wellbeing, household wellbeing, community relations, economic security and happiness. Each dimension is composed of sub‐dimensions; for example, physical strength included being able to space births, being able to do physical work, being free from diseases and having enough food.

The dimensions and sub‐dimensions are designed such that they can be used in a disaggregated way as a dashboard for tracking progress. However, to be used as an evaluative tool, for example, alongside a trial, they are more useful if aggregated into a single metric. Greco (2013) compared four different methods of valuing and indexing the (sub‐)dimensions and found that the choice of method impacted on the results. Notably, the capability index with weights derived using a normative approach based on ‘experts' opinions’ (which in this case are the women themselves) was found to give very similar results to the index aggregated using equal weights for each dimension. Greco recommends that researchers always make explicit the criteria used for establishing the weights and, when budget and time are limited, use equal weights.

The resulting index of women's capabilities was found to be a valid and reliable measure of quality of life, and it will be used to evaluate the impact of specific interventions targeting women's health in rural Malawi and in neighbouring Zambia. It is also being adapted for use in different country contexts.

#### 3.1.3 Progress and limitations of capability as an outcome measure

While the evaluation of the ACP in Thailand is an encouraging example of operationalising the capability approach as a measure of outcome in LMICs, ultimately, the evaluation used QALYs as the final outcome measure in the economic evaluation. This is partly driven by the strong health technology assessment programme in Thailand, Health Intervention and Technology Assessment Program (HITAP), and their comprehensive use of QALYs (not unlike NICE). The use of QALYs allowed the ICER of the ACP to be compared with a cost‐effectiveness benchmark defined by the National Health Security Office, which manages the HIV prevention programme in Thailand. If instead the evaluation had estimated the cost per unit of capability gained then without understanding the opportunity cost of a unit of capability (i.e. the cost‐effectiveness threshold), it would be difficult to assess the cost‐effectiveness of the programme. Without a greater understanding on the (monetary) value society places on capability, (this is not withstanding that we do not necessarily definitively know the monetary value of a QALY) using capability as an outcome measure essentially returns us to the realm of CEA.

Notably, the ACP evaluation used the OCAP‐18 instrument, which was developed in the UK, although they did test its translation, and it was developed for use in a public health setting. The ICECAP instruments are also UK centric, but they have been used in the Netherlands (Makai *et al*., 2012). Lorgelly (2014) argues that capabilities, unlike HRQoL measures, need to be culturally specific; translation and cultural adaptation may not be appropriate if the capabilities contained in the instrument do not encompass the capabilities of importance to the population of interest. Greco's (2013) work in Malawi avoids this criticism, as it produced an instrument that measures the quality of life of Malawian women. The latter approach is very resource/research intensive, and one must necessarily assess the trade‐offs of developing and using a population‐specific measure over using an ‘over the shelf’ approach.

### 3.2 Subjective wellbeing

Subjective wellbeing measures have gained popularity following the publications of a number of influential reports (Stiglitz, 2009, Helliwell *et al*., 2013) and the release of detailed guidelines by the OECD (2013) to standardise data collection across high‐income countries. The SWB approach fits with a traditional welfarist view of the world where individuals are regarded as the best judge of their own conditions, and the aim of public policy is to maximise the sum of everybody's happiness (or utility).

After spending much of his earlier career measuring HRQoL and employing standard valuation approaches, Dolan (1998) begun to question whether health states should be valued according to hypothetical preferences of the general public (as is the case with multi‐attribute utility instruments and, notably, the ICECAP measures) or based on subjective and experienced wellbeing. Dolan *et al*. (2005) reviewed a number of indices of wellbeing in order to understand the influences on SWB and application to policymaking, including income and life satisfaction questions, and found that some indices, such as income, offered an incomplete picture of wellbeing, which implies that additional measurement is required.

There are several different ways to measure SWB. Some measures focus on experienced happiness (as is Dolan's own preference), others focus on the evaluation of life, or life satisfaction, and some consider both. There is, however, evidence that life satisfaction and happiness (or affect) are not measuring the same concept. For example, education is associated with higher life evaluation, but not with more happiness (Kahneman and Deaton, 2010). Happiness varies over the days of the week and is better at weekends; life evaluation is the same on all days of the week. Happiness appears to become saturated with income beyond a point, while life evaluation does not. Life evaluation is U‐shaped with age, but stress, worry and anger diminish steadily with age from quite young ages (Deaton and Stone, 2014).

While SWB measures are attractive because they are relatively easy to collect and because they ask directly about one's feelings and life self‐evaluation, they might suffer from adaptation bias. According to Sen, ‘objectively’ deprived people cannot see the extent of their own deprivation because they are used to it, or this is what they have always seen around them (Sen, 1982). The validity and usefulness of happiness measures in this respect, especially in low‐income countries, are a matter of ongoing debate.

Cross‐country data reported by the Gallup World Survey in the World Happiness Survey (Helliwell *et al*., 2013) shed light on the differences between SWB measures (and their relationship with income). The top five ‘most life‐satisfied’ countries are Denmark, Norway, Switzerland, the Netherlands and Sweden. The bottom countries are Rwanda, Burundi, Central African Republic, Benin and Togo. The gap between the top and the bottom is wide: in the top five countries, life satisfaction is 7.48 out of 10, which is over 2.5 times the 2.94 average life satisfaction in the bottom countries. However, the picture of happiness is different: Danes and Italians experience less happiness than Pakistanis or Nepalese, and there is little or no correlation across countries between per‐capita GDP and happiness.

#### 3.2.1 The use of SWB in policy: evidence from the UK

Not unlike the measurement of capabilities, the measurement of SWB is an emerging area, and as such, there are few examples of its use in informing policy, and none in an LMIC setting. It is well known that Bhutan measures happiness (Burns, 2011), but at a national level, rather than a micro‐level. In the UK, Fujiwara (2013) has proposed the Wellbeing Valuation Approach, whereby wellbeing is valued using data on individual's SWB from large‐scale surveys (e.g. the British Households Panel Survey) to assess how non‐market goods or life events such as unemployment or illness impact on people's life satisfaction. For example, it is possible to estimate the monetary value of the SWB gain (or loss) because of the non‐market goods associated with the implementation of policy X, by looking at the impacts that policy X has on SWB: assume that the people affected by policy X have one index point higher life satisfaction (everything else being equal), the wellbeing valuation method then estimates, using the same dataset, how much extra income would also generate an equivalent one index point change in life satisfaction. If it is estimated that £1000 increases life satisfaction by one index point, then the monetary costs associated with the broader outcomes generated by policy X is equivalent to £1000 per person per year. This monetary valuation could, in theory, be compared with the cost of policy X, and in essence, a CBA be undertaken.

#### 3.2.2 Advantages and disadvantages of using SWB for policy in LMICs

A clear advantage of using SWB as an outcome measure is that it is relatively easy to measure. The OECD has a core set of questions pertaining to SWB (Box 1). They could easily be included in a survey to assess wellbeing; a baseline survey with follow‐up would allow an estimation of change in SWB, although like the metric of a unit of capability, this offers little benefit to a decision maker over and above what a CEA would deliver. Given the approach to valuing wellbeing (Fujiwara, 2013), it may be possible to undertake an analysis to understand the additional income, which is equivalent to a one‐point change in life satisfaction, which would then more readily inform a CBA and potentially aid decision certainty. This however requires a large dataset, although there are often many large studies undertaken in LMIC settings, like the Demographic and Health Surveys (www.dhsprogram.com).

**Box 1: OECD Core SWB questions (OECD, 2013)**

The following question asks how satisfied you feel, on a scale from 0 to 10. Zero means you feel ‘not at all satisfied’, and 10 means you feel ‘completely satisfied’.

A1 *Overall, how satisfied are you with life as a whole these days? [0–10]*

The following question asks how worthwhile you feel the things you do in your life are, on a scale from 0 to 10. Zero means you feel the things you do in your life are ‘not at all worthwhile’, and 10 means ‘completely worthwhile’.

A2 *Overall, to what extent do you feel the things you do in your life are worthwhile? [0–10]*

The following questions ask about how you felt yesterday on a scale from 0 to 10. Zero means you did not experience the feeling ‘at all’ yesterday, while 10 means you experienced the feeling ‘all of the time’ yesterday.

I will now read out a list of ways you might have felt yesterday.

A3 *How about happy? [0–10]*
A4 *How about worried? [0–10]*
A5 *How about depressed? [0–10]*

## 4 Conclusion

The use of traditional approaches to measuring outcomes of public health interventions in high‐income countries has of late been challenged. Recently, changes to NICE public health guidelines in the UK mean that capability can now be included as an outcome measure (National Institute for Health and Care Excellence, 2012). The inclusion of capability is because NICE believes that capability assessment offers something beyond health and that they value this additional evidence. Capabilities and SWB can offer value in LMIC settings also, but only if as outcomes they are valued. Ultimately, it comes down to the simple question: what do decision makers value? Do they value health, capabilities or SWB? Similarly, does the public, those individuals living in LMIC, value these outcomes? Future research should seek to understand this, perhaps following the approach of Borghi and Jan (2008), but rather than comparing health and non‐health outcomes, instead, compare health, capabilities and SWB. Given that LMICs are mostly devoid of fixed guidelines specifying the approach to evaluation, there are considerable gains to be made to research, evidence generation, decision science and, of course, population health, from embracing broader outcome measures.
